# Mapping the evolution and impact of microfluidic technology research on cancer diagnosis: A comprehensive bibliometric analysis from 2015 to 2024

**DOI:** 10.1097/MD.0000000000049910

**Published:** 2026-07-31

**Authors:** Xinxiang Su, Junjun Pu, Jinxia Liao, Jilin Li, Juanjuan Huang, Zhen Wu

**Affiliations:** a Guilin Medical University, Guilin, Guangxi, China; b Faculty of Basic Medical Science, Guilin Medical University, Guilin, Guangxi, China.

**Keywords:** bibliometrics, cancer diagnosis, microfluidic technology, visual analysis

## Abstract

**Background:**

To explore the research trends of microfluidic technology for cancer diagnosis from 2015 to 2024 using bibliometric and visualization methods (not a Preferred Reporting Items for Systematic Reviews and Meta-Analyses-compliant systematic review), and provide references for subsequent scientific research.

**Methods:**

Relevant literatures were retrieved from the Web of Science Core Collection, and analyzed using tools such as Origin 2018, R software, VOSviewer, and CiteSpace. The cooperation network, co - citation network, and keyword co - occurrence network was constructed.

**Results:**

A total of 897 literatures were included. The number of literatures first increased, then decreased, and then increased again, reaching a peak in 2023. China had the highest literature output (358 articles), but the international co - authorship rate was low (19%). *Biosensors & Bioelectronics* had high publication and citation numbers. The research hotspots were the isolation and detection of circulating tumor cells (with a focus on breast cancer, colorectal cancer, and lung cancer, accounting for 36%) and microfluidic analysis of exosomes (accounting for 24%). Keyword clustering involved microfluidic device materials, tumor cell detection, exosome applications, and cancer mechanism research. The research direction shifted from broad-based technology to precise applications for specific cancers and samples.

**Conclusion:**

This study clarified the research status and hotspots in this field. Microfluidic technology contributes to the early and accurate diagnosis of cancer. In the future, international cooperation should be strengthened to explore more potential of microfluidic technology in cancer diagnosis and improve the level of early diagnosis.

## 1. Introduction

Cancer poses a serious threat to human life and health.^[1]^ Early and accurate diagnosis is the key to improving patients’ survival rate and prognosis.^[2]^ Taking colorectal cancer as an example, if it is detected and treated in the early stage, the 5-year survival rate can reach over 90%, while when it is detected in the advanced stage, the survival rate may be <10%.^[3]^ However, traditional cancer diagnosis methods have many limitations. Tissue biopsy is an invasive procedure that can cause pain to patients and may also trigger complications such as infection and bleeding.^[4,5]^ Moreover, the obtained samples are limited, making it difficult to fully reflect the tumor characteristics.^[6]^ In the detection of early-stage cancers, the sensitivity and specificity of imaging examinations are also unsatisfactory, and it is easy to miss small lesions.^[7]^ Therefore, the development of more efficient, accurate, and minimally invasive cancer diagnosis technologies is urgent.

Microfluidic technology, as a cutting-edge technology, has great potential in the field of cancer diagnosis.^[8]^ Based on fluid manipulation at the microscale, it can precisely process and analyze biological samples.^[9,10]^ This technology has the advantages of high sensitivity, high throughput, and low sample consumption, bringing new ideas to cancer diagnosis.^[11]^ For example, in colorectal cancer, early detection via circulating tumor cells (CTCs) can significantly improve prognosis; similarly, CTC detection in breast cancer and lung cancer has shown promise in monitoring metastasis and guiding treatment^[12,13]^; in liquid biopsy, it can obtain tumor information from body fluids, promoting the development of noninvasive diagnosis.^[14,15]^ Microfluidic technology enables efficient capture of CTCs in these specific cancers, addressing the limitations of traditional methods

With the in - depth research of microfluidic technology in the field of cancer diagnosis, the number of relevant literatures has surged. A systematic analysis of these literatures helps to understand the research status, trends, and problems in this field. Bibliometrics is a powerful research tool. By quantitatively analyzing the characteristics of literatures such as the number of publications, citation situations, cooperation networks of authors and institutions, and keyword co-occurrences,^[16]^ it can reveal the development context, core forces, and hot directions of the research field.^[17]^

Notably, this study is a bibliometric analysis rather than a Preferred Reporting Items for Systematic Reviews and Meta-Analyses (PRISMA)-compliant systematic review. Pritchard defined bibliometrics as “the application of mathematical and statistical methods to the study of books and other means of communication” in 1969, and Hawkins further elaborated it as “the quantitative study of the characteristics of literature” in 2001.^[18]^ Today, bibliometrics has been widely applied in various disciplines to identify cutting - edge research and emerging trends.^[19]^ It has the advantages of an independent research scope and a comprehensive review of publications. Bibliometric mapping can also visualize the literature production patterns in scientific fields.^[20]^

Despite many studies on microfluidic technology in cancer diagnosis, comprehensive bibliometric analyses are lacking, hindering researchers from grasping the overall pattern, key achievements, and trends. This study fills this gap by bibliometrically analyzing 2015–2024 Web of Science literatures, mapping development, clarifying achievements, core forces, and hotspots to inform subsequent research, advance the technology, boost early accurate diagnosis, and improve patient survival and quality of life.

## 2. Materials and methods

### 2.1. Data collection

**Figure 1.**
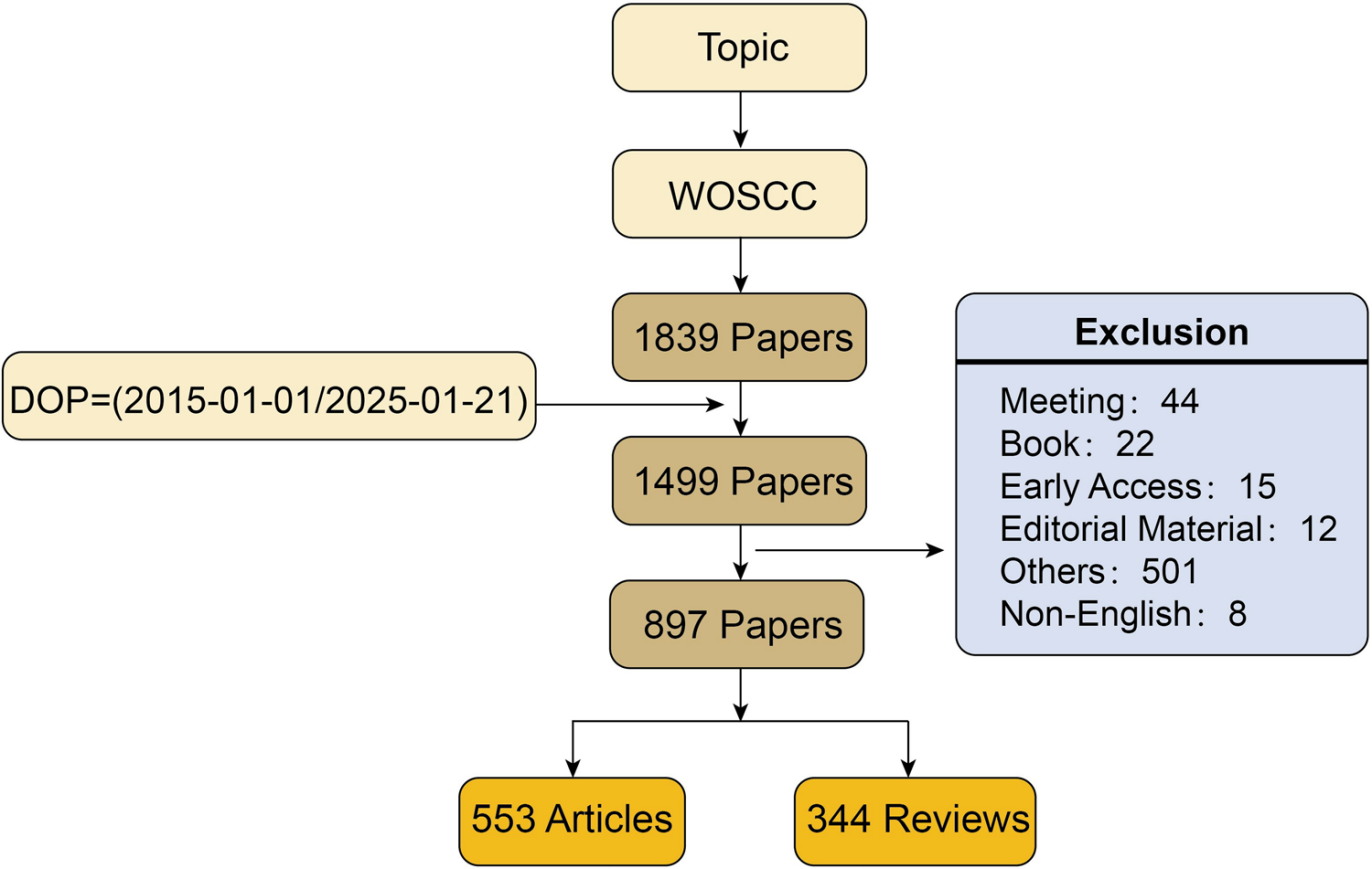
Schematic diagram elaborating the screening process for bibliometric data inclusion, which is not a PRISMA flow diagram (as this study is not a systematic review)

The data used in this study were retrieved and downloaded from the Web of Science Core Collection (WOSCC) (Guilin Medical University version) on January 21, 2025. The search formula was (((((TS = (Microfluidic technology OR Microfluidics)) AND TS = (Disease diagnosis OR Medical diagnosis)) AND TS = (Cancer OR Tumor)) AND DT = (Article OR Review)) AND LA = (English)) AND DOP = (2015 - 01 - 01/2025 - 01 - 21). The detailed process is shown in Figure 1. After removing duplicate literatures, the remaining retrieved articles were saved in plain text format, and their cited references were exported in the form of complete records. Protocol Registration: This bibliometric analysis does not require pre-registration. All methods are detailed for reproducibility.

### 2.2. Data analysis

Origin 2018 software was used to analyze the annual publication trends. The bibliometrix package (version 4.3.0)^[21]^ in R software (version 4.4.2), VOSviewer (version 1.6.18),^[22]^ and CiteSpace (version 6.3.1)^[23]^ were employed for the analysis and visualization of bibliometric data. The selection of these tools aims to ensure the accuracy and reliability of the data extraction and analysis process.

VOSviewer was used to draw visual charts such as the co - author networks of countries and institutions, co - citation analysis, and keyword co - occurrence networks. Specific criteria were applied in these analyses: For the co - author network analysis, a country must have at least 5 literatures and an institution must have at least 8 literatures to be included; in the co - citation analysis, only literature sources with at least 40 citations were considered; in the keyword co - occurrence analysis, keywords needed to appear in at least 25 literatures to be included, and the words “Microfluidics” and “Cancer” were deliberately excluded from this analysis. The journal impact factor (IF) data were sourced from the 2023 Journal Citation Reports.

The data analysis in this study follows bibliometric principles, focusing on quantitative characteristics of literatures rather than the qualitative synthesis of research findings. Since this is not a PRISMA-compliant systematic review, it does not involve steps required by PRISMA.

## 3. Results

### 3.1. Overview of research literature output on microfluidic technology and cancer diagnosis

**Figure 2.**
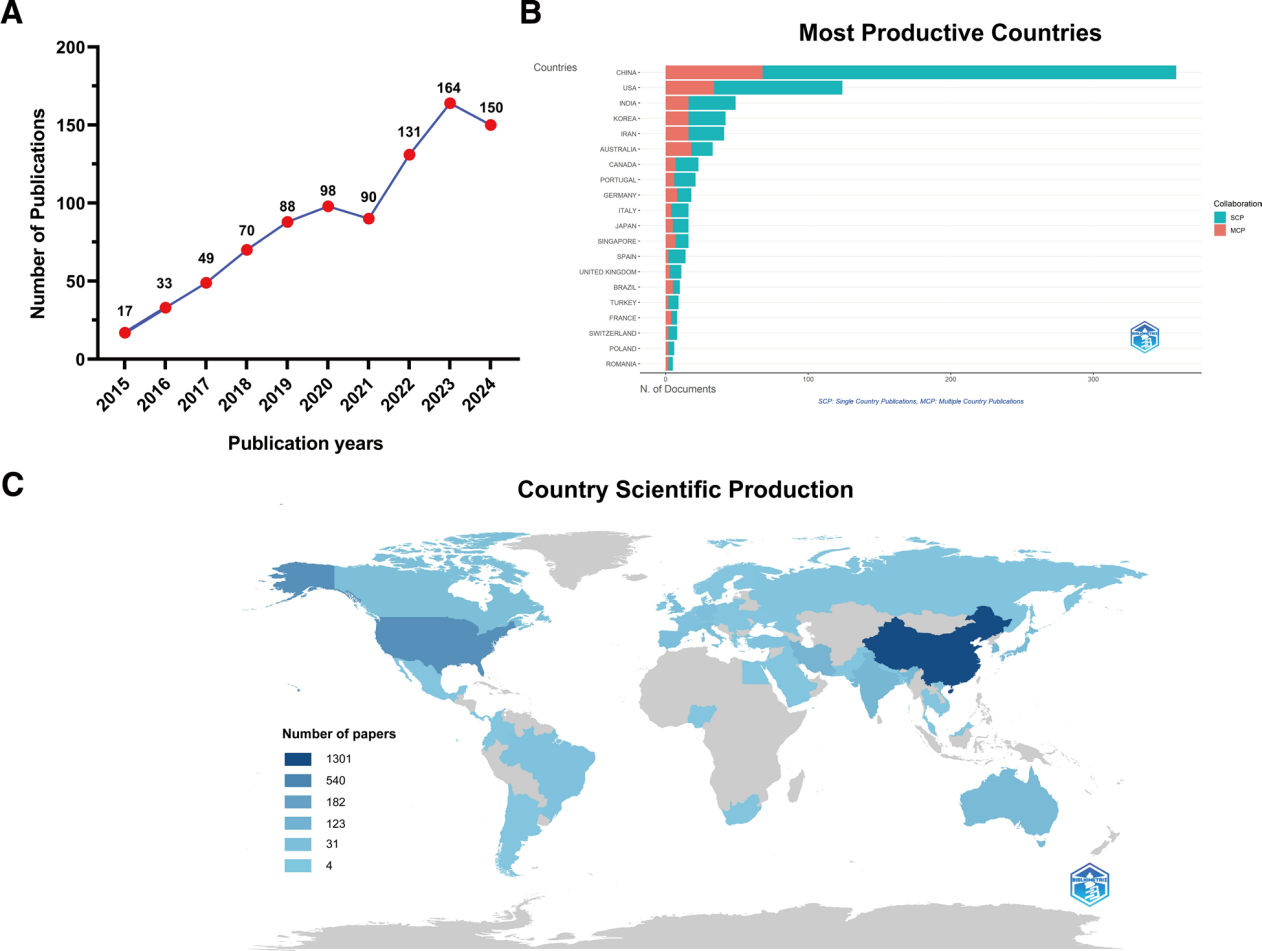
Trends of annual publications on microfluidic technology and cancer diagnosis research from 2015 to 2024. (A) Trend of annual publication output. (B) Distribution and cooperation of the countries where the corresponding authors are located. (C) Map of scientific production in various countries.

A total of 897 relevant literatures were identified from Web of Science. As shown in Figure 2A, publications grew from 2015 to 2020, slightly decreased to 90 in 2021, then surged to a peak of 164 in 2023, reflecting sustained interest and growth potential.

**Figure 3.**
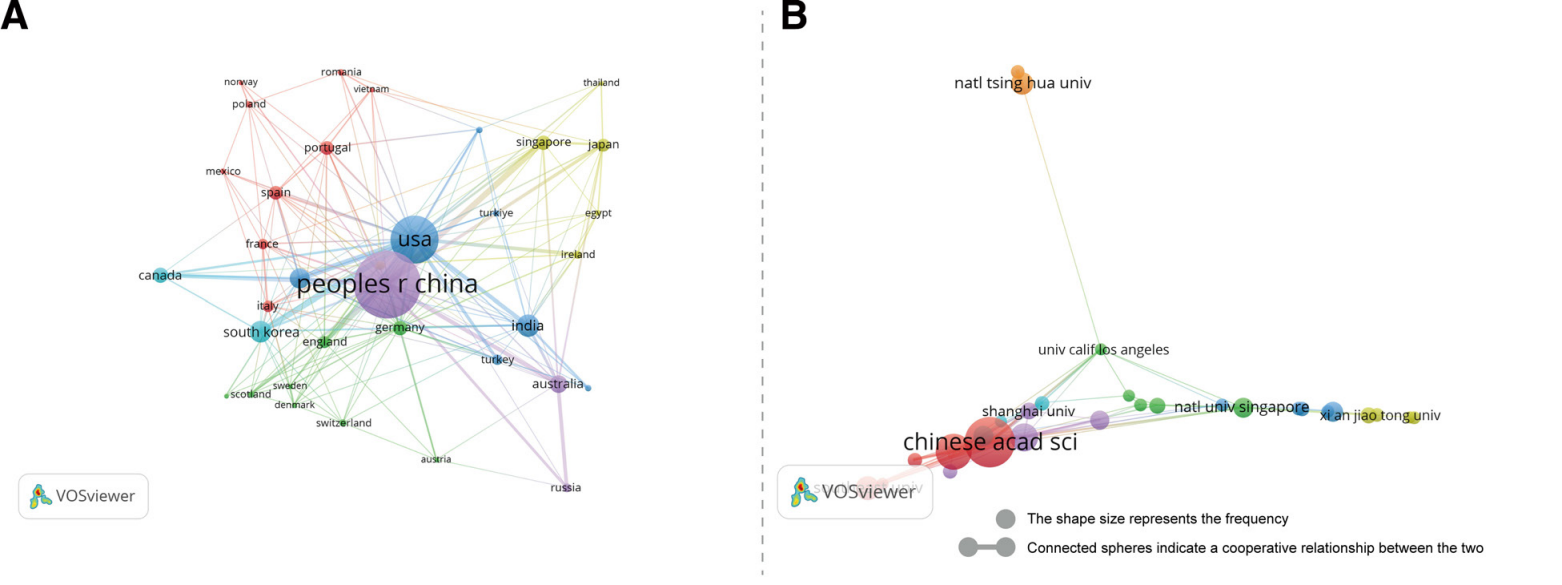
Distribution of Countries/Regions and Institutions in the Research of Microfluidic Technology and Cancer Diagnosis from 2015 to 2024. (A) Map of cooperation among different countries. (B) Map of cooperation among different institutions.

China led in outputs (358), followed by the United States (US) (124), India (49) and South Korea (42). The UAE (80%) and Australia (54.4%) had high international co-authorship rates, versus China (19%) and Spain (14.3%) (Figure 2B, 2C; Table 1). China (347 collaborations) and the US (28) were key in the international cooperation network (Figure 3A, 3B; Table 2).

**Table 1:** Most relevant countries by corresponding authors in microfluidic technology and cancer diagnosis research.

Country	Articles	SCP	MCP	Articles %	MCP %
China	358	290	68	39.9	19
USA	124	90	34	13.8	27.4
India	49	33	16	5.5	32.7
Korea	42	26	16	4.7	38.1
Iran	41	25	16	4.6	39
Australia	33	15	18	3.7	54.5
Canada	23	16	7	2.6	30.4
Portugal	21	15	6	2.3	28.6
Germany	18	10	8	2	44.4
Italy	16	12	4	1.8	25
Japan	16	11	5	1.8	31.3
Singapore	16	9	7	1.8	43.8
Spain	14	12	2	1.6	14.3
United Kingdom	11	8	3	1.2	27.3
Brazil	10	5	5	1.1	50
Turkey	9	7	2	1	22.2
France	8	4	4	0.9	50
Switzerland	8	6	2	0.9	25
Poland	6	4	2	0.7	33.3
Romania	5	3	2	0.6	40
Russia	5	4	1	0.6	20
U Arab Emirates	5	1	4	0.6	80
Belgium	4	2	2	0.4	50
Egypt	4	3	1	0.4	25
Ireland	4	2	2	0.4	50

MCP % = MCP/Articles, MCP = Multiple Country Publications, SCP = Single Country Publications.

**Table 2:** Most relevant affiliations in microfluidic technology and cancer diagnosis research.

Affiliation	Articles(n)
Chinese Academy Of Sciences	137
University Of California System	61
National Tsing Hua University	52
Tabriz University Of Medical Science	43
University Of Chinese Academy Of Sciences, Cas	43
National University Of Singapore	41
Shanghai Jiao Tong University	32
Southeast University - China	31
National Cheng Kung University	30
Fudan University	29
Indian Institute Of Technology System (Iit System)	28
Xiamen University	28
Nagoya University	25
Harvard University	24
University Of Queensland	24
City University Of Hong Kong	22
Yonsei University	22
University System Of Ohio	20
University Of Connecticut	19
Yangzhou University	18
Centre National De La Recherche Scientifique (Cnrs)	17
Shanghai Institute Of Microsystem And Information Technology, Cas	17
University Of California Los Angeles	17
Wuhan University	17
Xi’an Jiaotong University	17

Major funding institutions included the Chinese Academy of Sciences, University of California System, etc, with most funding from China. National Natural Science Foundation of China (NSFC) topped funding records (237), followed by National Institutes of Health (NIH) (75) and US Department of Health and Human Services (75) (Table 3), providing insights for global research funding dynamics.

**Table 3:** The top 20 funding agencies in the field of microfluidic technology and cancer diagnosis research.

Funding Agencies	Record Count
National Natural Science Foundation Of China Nsfc	237
National Institutes Of Health Nih Usa	75
United States Department Of Health Human Services	75
National Key Research Development Program Of China	39
Fundamental Research Funds For The Central Universities	34
National Science Foundation Nsf	34
Nih National Cancer Institute Nci	33
National Research Foundation Of Korea	29
Ministry Of Science And Technology Taiwan	25
European Union Eu	24
National Key R D Program Of China	22
Fundacao Para A Ciencia E A Tecnologia Fct	18
Natural Sciences And Engineering Research Council Of Canada Nserc	17
China Postdoctoral Science Foundation	16
Nih National Institute Of Biomedical Imaging Bioengineering Nibib	15
Science Technology Commission Of Shanghai Municipality Stcsm	15
Chinese Academy Of Sciences	14
Ministry Of Education Culture Sports Science And Technology Japan Mext	14
Australian Research Council	13
Natural Science Foundation Of Jiangsu Province	13

### 3.2. Journals and co-cited journals

**Figure 4.**
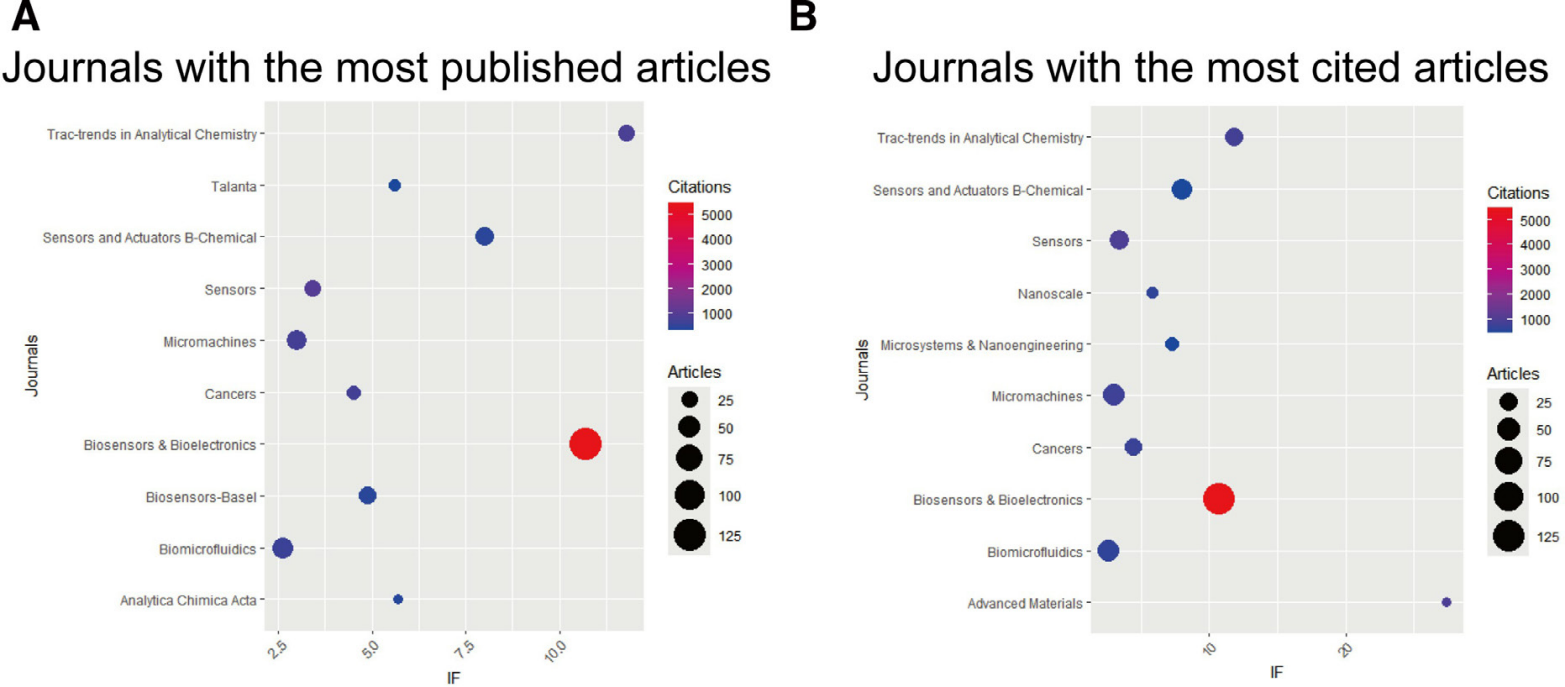
Journals with the Largest Number of Published Articles and Journals with the Highest Number of Citations. (A) Journals with the largest number of published articles. (B) Journals with the highest number of citations.

Using Bibliometrix and ggplot2 in R, top journals by publications included Biosensors & Bioelectronics (125, IF = 10.7), Biomicrofluidics (41, IF = 2.6), Micromachines (38, IF = 3.0), and Sensors and Actuators B-Chemical (33, IF = 8.0) (Table 4, Figure 4A). By citations, Biosensors & Bioelectronics led (5490, IF = 10.7), followed by Sensors (974, IF = 3.4), Advanced Materials (954, IF = 27.4), Trac-Trends Anal. Chem. (822, IF = 11.8), and Micromachines (750, IF = 3.0) (Table 5, Figure 4B). Biosensors & Bioelectronics ranked consistently high, while others varied in publication count versus citation influence.

**Table 4:** Top 10 journals with the most published articles.

Journal	Articles	IF (2023)	Cites
Biosensors & Bioelectronics	125	10.7	5490
Biomicrofluidics	41	2.6	611
Micromachines	38	3.0	750
Sensors and Actuators B-Chemical	33	8.0	438
Biosensors-Basel	28	4.9	431
Sensors	27	3.4	974
Trac-trends in Analytical Chemistry	26	11.8	822
Cancers	22	4.5	710
Talanta	19	5.6	317
Analytica Chimica Acta	18	5.7	407

IF = Impact Factor.

**Table 5:** Top 10 journals with the most cited articles.

Journal	Cites	IF (2023)	Documents
Biosensors & Bioelectronics	5490	10.7	125
Sensors	974	3.4	27
Advanced Materials	954	27.4	8
Trac-trends in Analytical Chemistry	822	11.8	26
Micromachines	750	3.0	38
Cancers	710	4.5	22
Biomicrofluidics	611	2.6	41
Nanoscale	602	5.8	9
Sensors and Actuators B-Chemical	438	8.0	33
Microsystems & Nanoengineering	432	7.3	12

IF = Impact Factor.

**Figure 5.**
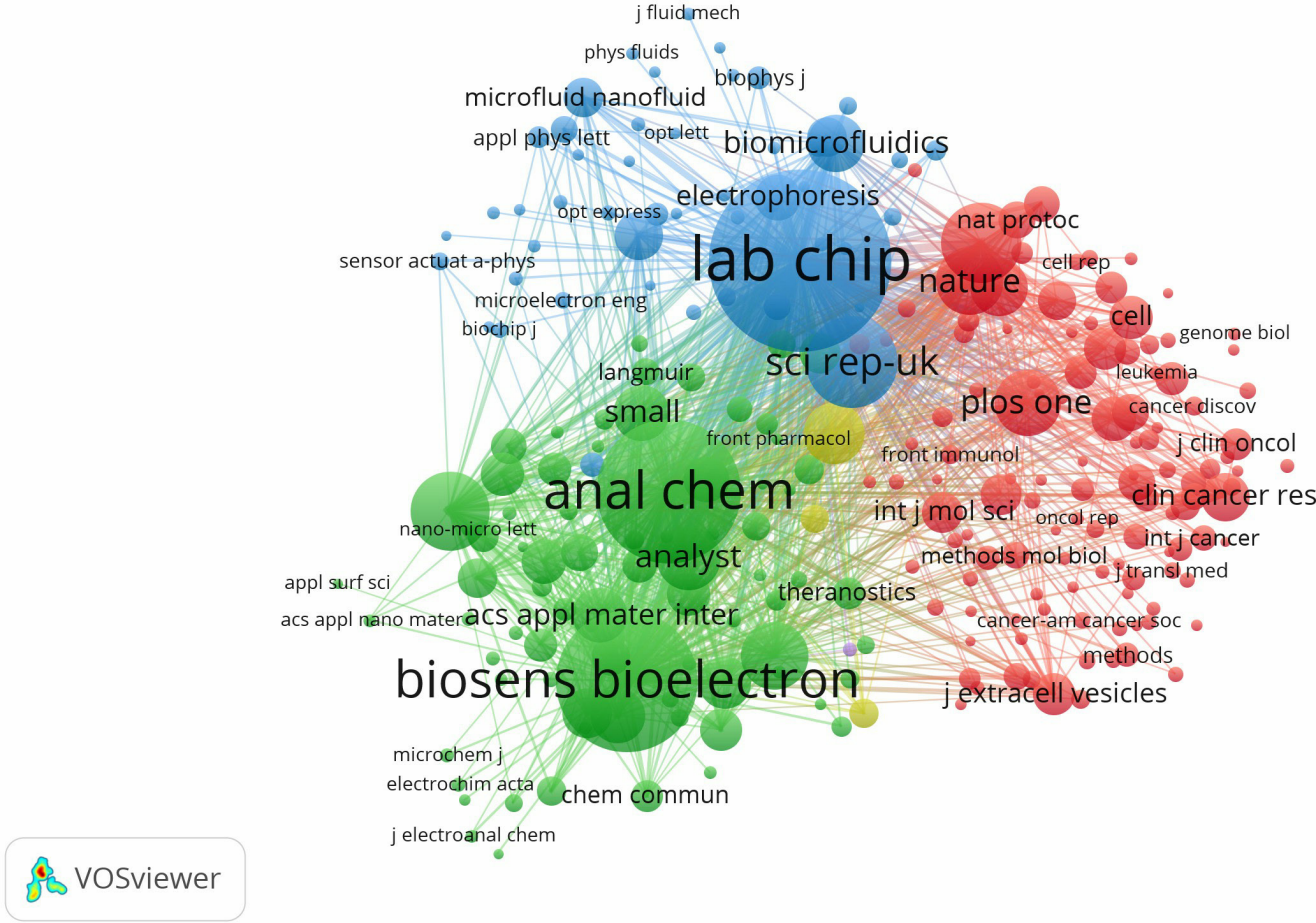
Co - cited Journals Involved in the Research of Microfluidic Technology and Cancer Diagnosis.

VOSviewer (v1.6.18) analyzed co-citations of 897 literatures across 200 journals, identifying Lab on a Chip, Anal. Chem., and Biosens. Bioelectron. as core hubs (Fig. 5), pivotal in guiding the field. Notably, top-tier journals lack sufficient related studies, emphasizing the need for deeper, higher-quality research.

### 3.3. Highly cited references and citation bursts

Using R’s bibliometrix (v4.3.0), we identified the top 20 most-cited references (≥147 citations across 14 journals; Table 6), with no dominant journal. Top 3 focused on extracellular vesicles, CRISPR/Cas13a biosensors, and impedance measurements, highlighting microfluidics’ potential in noninvasive, rapid cancer diagnosis.

**Table 6:** Top 20 cited references related to microfluidic technology and cancer diagnosis research.

Paper	DOI	Total Citations	TC per Year
RAMIREZ MI, 2018, NANOSCALE	10.1039/c7nr08360b	371	46.38
BRUCH R, 2019, ADV MATER	10.1002/adma.201905311	349	49.86
XU YC, 2016, BIOSENS BIOELECTRON	10.1016/j.bios.2015.10.027	325	32.50
POTTIER C, 2020, CANCERS	10.3390/cancers12030731	301	50.17
CHEN JC, 2022, FRONT BIOENG BIOTECH	10.3389/fbioe.2021.811971	290	72.50
CAO L, 2017, BIOSENS BIOELECTRON	10.1016/j.bios.2016.09.082	210	23.33
HYUN KA, 2016, ONCOTARGET	10.18632/oncotarget.8250	204	20.40
MAHATO K, 2017, BIOSENS BIOELECTRON	10.1016/j.bios.2017.05.001	200	22.22
PANDEY CM, 2018, BIOTECHNOL J	10.1002/biot.201700047	189	23.63
SINGH AT, 2018, SENSORS-BASEL	10.3390/s18092838	185	23.13
WANG Y, 2019, BIOSENS BIOELECTRON	10.1016/j.bios.2019.04.032	181	25.86
GUO B, 2019, ADV MATER	10.1002/adma.201902504	176	25.14
CHEN H, 2019, CLIN CHIM ACTA	10.1016/j.cca.2019.03.008	167	23.86
YANG Y, 2020, SMALL METHODS	10.1002/smtd.201900451	153	25.50
CUI FY, 2019, J ELECTROCHEM SOC	10.1149/2.0252003JES	152	21.71
ANTFOLK M, 2017, ANAL CHIM ACTA	10.1016/j.aca.2017.02.017	151	16.78
DEVI RV, 2015, BIOSENS BIOELECTRON	10.1016/j.bios.2015.01.066	149	13.55
CHEN BY, 2019, CLIN CHIM ACTA	10.1016/j.cca.2019.02.021	148	21.14
CABALLERO D, 2017, BIOMATERIALS	10.1016/j.biomaterials.2017.10.005	147	16.33
NOSRATI R, 2017, NAT REV UROL	10.1038/nrurol.2017.175	147	16.33

DOI = Digital Object Identifier, TC = Total Citations.

**Figure 6.**
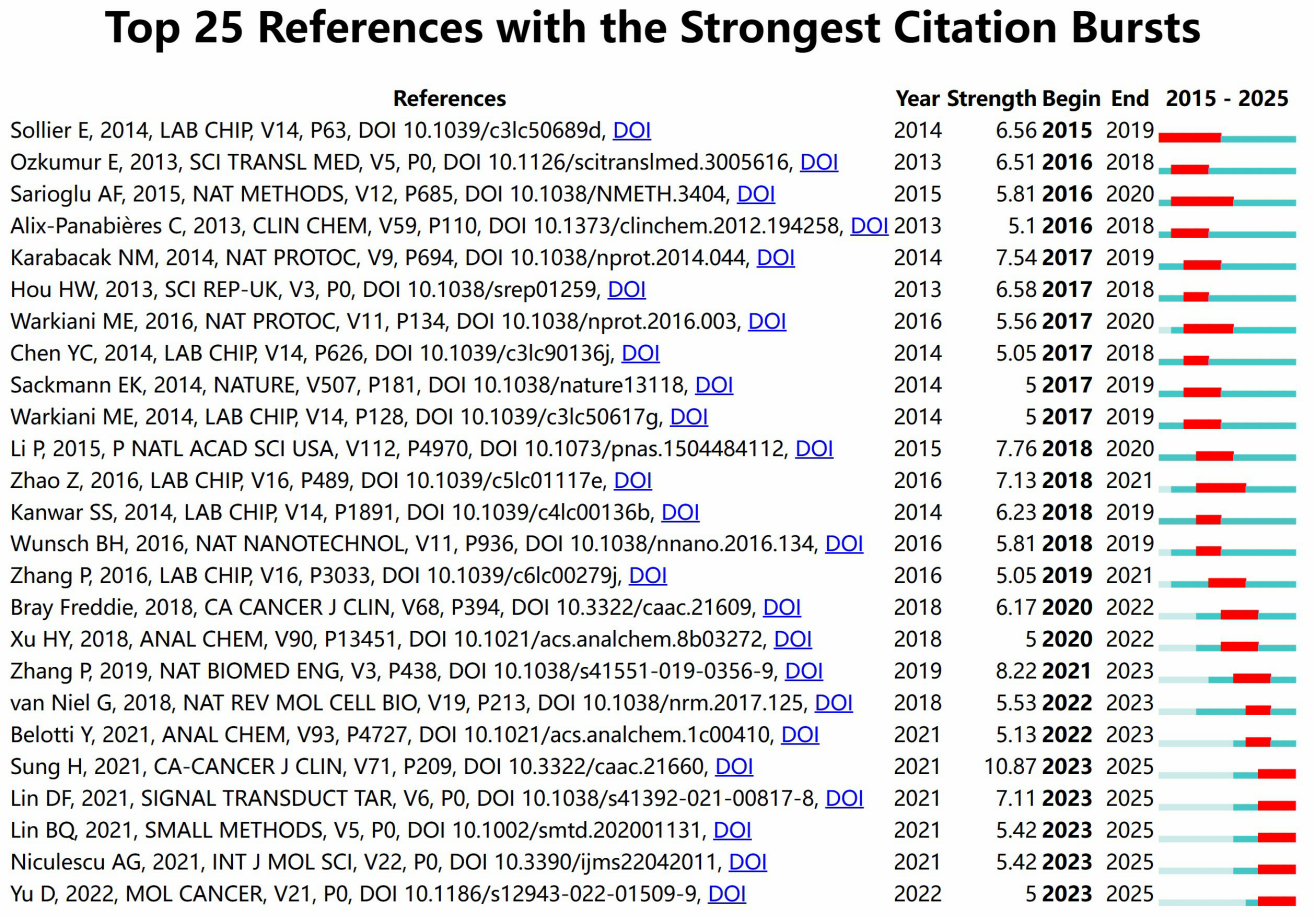
The top 25 references with the strongest burst strength in the research of microfluidic technology and cancer diagnosis.

CiteSpace (top 21, state count = 2, duration ≥ 2) revealed 25 citation bursts (Fig. 6), led by “Global Cancer Statistics 2020” (strength = 10.87), “3D-nanopatterned chip for exosome detection” (8.22), and “acoustic CTC separation” (7.76). Frontier bursts included exosome liquid biopsy and microfluidic device reviews.

Correlating 25 citations (Fig. 6) with Annex 1, Supplemental Digital Content 1 showed “CTC separation/detection” (36%) and “exosome analysis” (24%) as hotspots; CTC studies focused on breast (14%), colorectal (12%), and lung (10%) cancers.

Microfluidics’ high sensitivity/throughput aids early diagnosis but faces standardization and clinical translation challenges, requiring further research to advance early detection and precision medicine.

### 3.4. Keyword clustering and evolution

VOSviewer identified 4038 keywords, with top 20 (≥43 occurrences) including “circulating tumor-cells” (137), “liquid biopsy” (97), and “exosomes” (75) (Table 7).

**Table 7:** Top 20 keywords related to microfluidic technology and cancer diagnosis research.

Rank	Words	Occurrences
1	circulating tumor - cells	137
2	liquid biopsy	97
3	Separation	82
4	Biomarkers	78
5	Exosomes	75
6	Chip	74
7	Blood	72
8	circulating tumor cells	72
9	Diagnosis	71
10	extracellular vesicles	69
11	breast - cancer	63
12	microfluidic chip	63
13	Device	58
14	nanoparticles	58
15	Biosensor	57
16	Capture	53
17	label – free	53
18	Platform	51
19	Size	47
20	microfluidic	43

**Figure 7.**
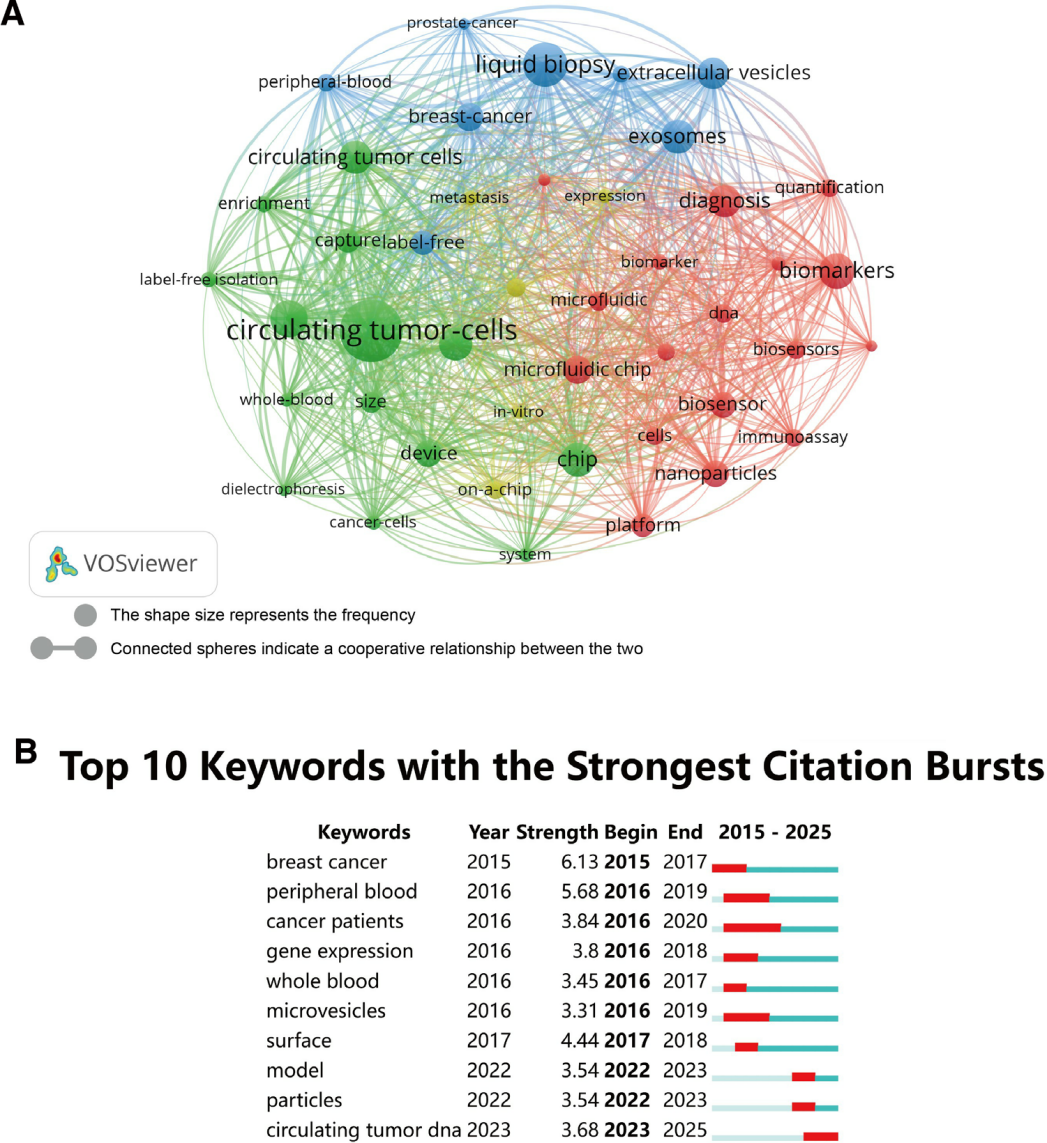
Keyword analysis diagrams of the research on microfluidic technology and cancer diagnosis. (A) Keyword co - occurrence map of the literatures related to the research on microfluidic technology and cancer diagnosis. (B) The top 10 keywords with the strongest burst strength in the research on microfluidic technology and cancer diagnosis.

46 keywords (≥25 occurrences) formed 4 clusters (Fig. 7A): red (17 terms: “microfluidic chip,” “nanoparticles,” etc), green (14 terms: “CTCs,” “label-free isolation,” etc), blue (8 terms: “exosomes,” “peripheral-blood,” etc), yellow (5 terms: “metastasis,” etc) (Annex 2, Supplemental Digital Content 2). Citation bursts (2022 to 2025) included “model,” “particles,” “circulating tumor dna” (Fig. 7B).

**Figure 8.**
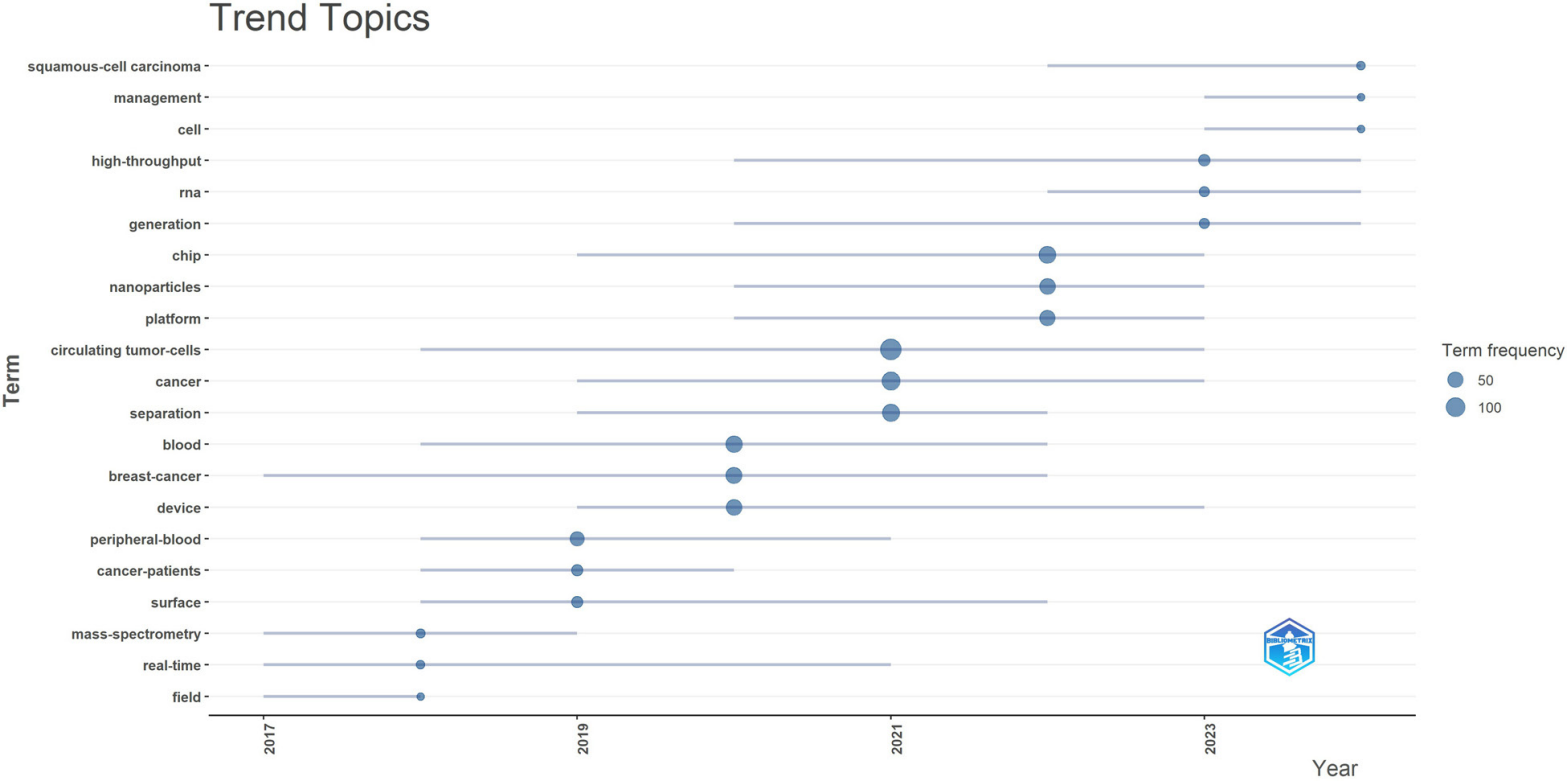
Trend - based themes of the research on microfluidic technology and cancer diagnosis.

Bibliometrix-generated hot-topic map (Fig. 8) showed research shifts: 2017 to 2023 from broad technologies (“field,” “mass-spectrometry”) to specific applications (“surface,” “cancer-patients”), with recent focus on “breast-cancer,” “blood,” “separation.” Key topics like “CTCs” and “nanoparticles” remain prominent, with future directions in multidisciplinary integration and clinical expansion.

## 4. Discussion

### 4.1. General information

This study bibliometrically analyzed 897 2015–2024 literatures on microfluidic cancer diagnosis, showing fluctuating publication trends with growing interest and potential. China led in outputs (358) but had low international co-authorship (19%), lagging the UAE (80%) and Australia (54.4%); Chinese Academy of Sciences and NSFC (237 funds) were prominent. Biosensors & Bioelectronics stood out (125 articles, 5490 citations); other journals varied in publications versus citations, reflecting diverse focuses.

### 4.2. Hotspots and development trends

This study integrates highly cited literature, citation bursts, keyword clustering, and topic trends to explore research hotspots and frontier directions of microfluidic technology in cancer diagnosis.

Highly cited literature focuses on microfluidic technology for noninvasive, rapid, accurate cancer diagnosis, particularly nucleic acid detection and biosensing.^[24–27]^ The research achievements presented in numerous literatures fully demonstrate the great potential of microfluidic technology in these application scenarios. Among them, “separation and detection of CTCs”^[28,29]^ and “microfluidic analysis of extracellular vesicles (EVs) in cancer diagnosis”^[30,31]^ occupy prominent positions, accounting for 36% and 24% respectively in the research hotspots.

Microfluidic CTC detection provides key insights for specific cancer management, overcoming traditional diagnostic limits. In early colorectal cancer, it captures rare CTCs for noninvasive monitoring with biosensor-aided molecular profiling. For triple-negative breast cancer, inertial microfluidics isolates CTCs to guide treatment; in CA19-9-negative pancreatic cancer, it boosts detection sensitivity; in non-small-cell lung cancer, multimodal capture aids heterogeneous CTC detection for monitoring. Translation faces standardization, CTC subtype relevance, and cost issues. Future work should optimize cancer-specific devices, integrate multi-analyte detection, validate regulation, and enhance international collaboration to bridge lab-clinic gaps, advancing early diagnosis and personalized monitoring.

From the results of keyword clustering analysis, the research focus is further refined into several key directions: The research on microfluidic devices and materials centered around “microfluidic chips,” “nanoparticles,” etc, aims to enhance performance indicators such as the sensitivity and specificity of cancer detection through material innovation and device optimization.^[32,33]^ The research on the detection and separation of “CTCs,” as a crucial link in early - stage cancer diagnosis, provides important evidence for clinicians to detect cancer in a timely manner.^[34]^ The exploration of the application of “extracellular vesicles” in cancer diagnosis is committed to discovering new biomarkers, opening up new avenues for early - stage cancer screening and precise diagnosis.^[35,36]^ The research on cancer metastasis and gene expression mechanisms helps researchers deeply understand the development process of cancer, laying a foundation for the development of more effective diagnostic and treatment strategies.^[37,38]^ These research hotspots are widely present in various existing related studies and are the core areas that current researchers focus on.

Development trends shifted from early broad exploration of basic technologies and general detection methods to targeting specific cancers and refined sample processing. Microfluidic technology is optimized for specific diagnostic needs, e.g., targeted methods for breast cancer (based on its unique cell characteristics) have improved early diagnosis accuracy.^[39,40]^ At the same time, sample processing is becoming increasingly important in cancer diagnosis research. How to efficiently and accurately obtain cancer - related information from various samples such as blood and tissue fluid has become the key focus of current research.^[41,42]^ Looking ahead, multi - disciplinary integration will become the core driving force for the continuous development of microfluidic technology in the field of cancer diagnosis.^[43]^ Through the collaborative innovation of materials science, biology, medical engineering, and other disciplines, it is expected to develop more advanced microfluidic devices, further enhancing the accuracy and efficiency of cancer diagnosis.^[44]^ In addition, with the continuous deepening of the concept of precision medicine in the medical field, microfluidic technology will also follow this trend and move towards personalized diagnosis.^[45]^ By precisely grasping the individual differences of patients, personalized diagnostic plans can be tailored for each cancer patient, thus better meeting the actual clinical needs and providing more solid technical support for the early diagnosis and effective treatment of cancer patients.

### 4.3. Advantages and limitations

This study comprehensively and systematically analyzed the research status of microfluidic technology in the field of cancer diagnosis by means of various bibliometric tools and methods. The research results provide strong support for researchers to sort out the development context of this field and grasp the research hotspots and trends, helping researchers to explore new research directions and reasonably plan research projects.

It should not be overlooked that this study has certain limitations Firstly, our data was solely sourced from the Web of Science (WoS) database, which might have led to the omission of some publications. However, the WoS database is a widely recognized high-quality digital literature database and one of the top choices for bibliometric analysis,^[46–48]^ thus ensuring the reliability of the data source. Secondly, only English publications were analyzed, which might introduce a source bias. Despite these limitations, this study still comprehensively presents the overall situation, hotspots, and research trends in this field.

## 5. Conclusion

This bibliometric analysis reviewed 2015–2024 research on microfluidic technology in cancer diagnosis, offering key insights:

a. Literature & Geography: Publications grew 2015 to 2020, dipped to 90 in 2021, then peaked at 164 in 2023. China led with 358 papers but had low international co-authorship (19%), lagging the UAE (80%) and Australia (54.4%).
b. Core Journals: *Biosensors & Bioelectronics* stood out (125 papers, 5490 citations). Others varied in publication count versus citation impact, reflecting diverse focuses.
c. Hotspots: Highly cited studies focused on noninvasive, rapid diagnosis (e.g., nucleic acid detection). “CTC separation/detection” (36%) and “exosome analysis” (24%) were dominant.
d. Keyword Clusters: Four clusters emerged: microfluidic devices/materials (red), CTC detection/separation (green), exosome applications (blue), and cancer metastasis/genetics (yellow).
e. Trends: The research trend has shifted from broad technical detection to specific cancers and refined sample processing. In CTC detection, microfluidic technology is customized and optimized according to the characteristics of CTCs in different cancers, and will focus more on clinical integration in the future to promote its transformation into routine diagnosis.

In summary, this study outlines achievements, hotspots, and trends, guiding advances in early cancer diagnosis and patient care.

## Acknowledgments

We were grateful to everyone who participated in the study and helped with data analyses and preparation of the manuscript. We would also like to thank the editor and reviewers for their valuable insights and assistance.

## Author contributions

**Conceptualization:** Zhen Wu.

**Data curation:** Xinxiang Su, Zhen Wu.

**Formal analysis:** Xinxiang Su, Zhen Wu.

**Investigation:** Zhen Wu.

**Methodology:** Xinxiang Su, Zhen Wu.

**Resources:** Xinxiang Su, Junjun Pu, Jinxia Liao, Jilin Li, Juanjuan Huang.

**Software:** Xinxiang Su, Junjun Pu, Jinxia Liao, Jilin Li, Juanjuan Huang.

**Supervision:** Junjun Pu, Jinxia Liao, Jilin Li, Juanjuan Huang.

**Writing – original draft:** Xinxiang Su.

**Writing – review & editing:** Xinxiang Su.





## References

[1] Arndt MB Abate YH Abbasi-Kangevari M. Global, regional, and national progress towards the 2030 global nutrition targets and forecasts to 2050: a systematic analysis for the Global Burden of Disease Study 2021. The Lancet. 2024;404:2543–83. 10.1016/S0140-6736(24)01821-X PMC11703702 39667386

[2] Song B Liang R. Integrating artificial intelligence with smartphone-based imaging for cancer detection in vivo. Biosens Bioelectron. 2025;271:116982. 39616900 10.1016/j.bios.2024.116982 PMC11789447

[3] Huang S Yang J Shen N Xu Q Zhao Q. Artificial intelligence in lung cancer diagnosis and prognosis: current application and future perspective. Semin Cancer Biol. 2023;89:30–7. 36682439 10.1016/j.semcancer.2023.01.006

[4] Ding Z Wang N Ji N Chen ZS. Proteomics technologies for cancer liquid biopsies. Mol Cancer. 2022;21:53. 35168611 10.1186/s12943-022-01526-8 PMC8845389

[5] Guo Y Zhang Y Gerhard M. Effect of helicobacter pylori on gastrointestinal microbiota: a population-based study in Linqu, a high-risk area of gastric cancer. Gut. 2020;69:1598–607. 31857433 10.1136/gutjnl-2019-319696 PMC7456744

[6] Mirza AN Mirza NQ Vlastos G Singletary SE. Prognostic factors in node-negative breast cancer: a review of studies with sample size more than 200 and follow-up more than 5 years. Ann Surg. 2002;235:10–26. 11753038 10.1097/00000658-200201000-00003 PMC1422391

[7] Lu G Little JV Wang X. Detection of head and neck cancer in surgical specimens using quantitative hyperspectral imaging. Clin Cancer Res. 2017;23:5426–36. 28611203 10.1158/1078-0432.CCR-17-0906 PMC5649622

[8] Zhao L Wang H Fu J. Microfluidic-based exosome isolation and highly sensitive aptamer exosome membrane protein detection for lung cancer diagnosis. Biosens Bioelectron. 2022;214:114487. 35780540 10.1016/j.bios.2022.114487

[9] Luo T Fan L Zhu R Sun D. Microfluidic single-cell manipulation and analysis: methods and applications. Micromachines. 2019;10:104. 30717128 10.3390/mi10020104 PMC6412357

[10] Liu X Zheng X. Microfluidic-based electrical operation and measurement methods in single-cell analysis. Sensors (Basel, Switzerland). 2024;24:6359. 39409403 10.3390/s24196359 PMC11478560

[11] Deng Z Wu S Wang Y Shi D. Circulating tumor cell isolation for cancer diagnosis and prognosis. EBioMedicine. 2022;83:104237. 36041264 10.1016/j.ebiom.2022.104237 PMC9440384

[12] Vaidyanathan R Soon RH Zhang P Jiang K Lim CT. Cancer diagnosis: from tumor to liquid biopsy and beyond. Lab Chip. 2018;19:11–34. 30480287 10.1039/c8lc00684a

[13] Myung JH Hong S. Microfluidic devices to enrich and isolate circulating tumor cells. Lab Chip. 2015;15:4500–11. 26549749 10.1039/c5lc00947b PMC4664604

[14] Marassi V Giordani S Placci A. Emerging microfluidic tools for simultaneous exosomes and cargo biosensing in liquid biopsy: new integrated miniaturized FFF-assisted approach for colon cancer diagnosis. Sensors (Basel, Switzerland). 2023;23:9432. 38067805 10.3390/s23239432 PMC10708636

[15] Akgönüllü S Bakhshpour M Pişkin AK Denizli A. microfluidic systems for cancer diagnosis and applications. Micromachines. 2021;12:1349. 34832761 10.3390/mi12111349 PMC8619454

[16] Ling LX Ouyang Y Hu Y. Research trends on nanomaterials in gastric cancer: a bibliometric analysis from 2004 to 2023. J Nanobiotechn. 2023;21:248. 10.1186/s12951-023-02033-8 PMC10394877 37533041

[17] Zhou X Li Z Zheng T. Review of global sanitation development. Environ Int. 2018;120:246–61. 30103124 10.1016/j.envint.2018.07.047 PMC6192828

[18] Kokol P Blažun Vošner H Završnik J. Application of bibliometrics in medicine: a historical bibliometrics analysis. Health Info Libr J. 2021;38:125–38. 31995273 10.1111/hir.12295

[19] Liu X Zhao S Tan L. Frontier and hot topics in electrochemiluminescence sensing technology based on CiteSpace bibliometric analysis. Biosens Bioelectron. 2022;201:113932. 35065388 10.1016/j.bios.2021.113932

[20] Yu Y Wang S Yu P. A bibliometric analysis of emerging contaminants (ECs) (2001-2021): evolution of hotspots and research trends. Sci Total Environ. 2024;907:168116. 37884150 10.1016/j.scitotenv.2023.168116

[21] Aria M Cuccurullo C. bibliometrix: an R-tool for comprehensive science mapping analysis. J Informetr. 2017;11:959–75.

[22] van Eck NJ Waltman L. Software survey: VOSviewer, a computer program for bibliometric mapping. Scientometrics. 2010;84:523–38. 20585380 10.1007/s11192-009-0146-3 PMC2883932

[23] Chen CM. CiteSpace II: detecting and visualizing emerging trends and transient patterns in scientific literature. J Am Soc Inf Sci Technol. 2006;57:359–77.

[24] Yin J Suo Y Zou Z. Integrated microfluidic systems with sample preparation and nucleic acid amplification. Lab Chip. 2019;19:2769–85. 31365009 10.1039/c9lc00389d PMC8876602

[25] Wang X Hong XZ Li YW. Microfluidics-based strategies for molecular diagnostics of infectious diseases. Mil Med Res. 2022;9:11. 35300739 10.1186/s40779-022-00374-3 PMC8930194

[26] Farshchi F Hasanzadeh M. Microfluidic biosensing of circulating tumor cells (CTCs): recent progress and challenges in efficient diagnosis of cancer. Biomed Pharmacother = Biomedecine & pharmacotherapie. 2021;134:111153. 33360045 10.1016/j.biopha.2020.111153

[27] Adampourezare M Dehghan G Hasanzadeh M Feizi MAH. Application of lateral flow and microfluidic bio-assay and biosensing towards identification of DNA-methylation and cancer detection: recent progress and challenges in biomedicine. Biomed Pharmacother = Biomedecine & pharmacotherapie. 2021;141:111845. 34175816 10.1016/j.biopha.2021.111845

[28] Li C He W Wang N. Application of microfluidics in detection of circulating tumor cells. Front Bioeng Biotechnol. 2022;10:907232. 35646880 10.3389/fbioe.2022.907232 PMC9133555

[29] Zhu S Jiang F Han Y Xiang N Ni Z. Microfluidics for label-free sorting of rare circulating tumor cells. Analyst. 2020;145:7103–24. 33001061 10.1039/d0an01148g

[30] Abreu CM Costa-Silva B Reis RL Kundu SC Caballero D. Microfluidic platforms for extracellular vesicle isolation, analysis and therapy in cancer. Lab Chip. 2022;22:1093–125. 35253032 10.1039/d2lc00006g

[31] Zhand S Goss DM Cheng YY Warkiani ME. Recent advances in microfluidics for nucleic acid analysis of small extracellular vesicles in cancer. Adv Healthcare Mater. 2024;14:e2401295. 10.1002/adhm.202401295 39707658

[32] Cheng SJ Hsieh KY Chen SL. Microfluidics and nanomaterial-based technologies for circulating tumor cell isolation and detection. Sensors (Basel, Switzerland). 2020;20:1875. 32230996 10.3390/s20071875 PMC7180594

[33] Li Y Wong IY Guo M. Reciprocity of cell mechanics with extracellular stimuli: emerging opportunities for translational medicine. Small (Weinheim an der Bergstrasse, Germany). 2022;18:e2107305. 35319155 10.1002/smll.202107305 PMC9463119

[34] Zhu Z Zhang Y Zhang W. High-throughput enrichment of portal venous circulating tumor cells for highly sensitive diagnosis of CA19-9-negative pancreatic cancer patients using inertial microfluidics. Biosens Bioelectron. 2024;259:116411. 38781696 10.1016/j.bios.2024.116411

[35] Mousavi SM Mahdian SMA Ebrahimi MS. Microfluidics for detection of exosomes and microRNAs in cancer: state of the art. Mol Ther Nucleic Acids. 2022;28:758–91. 35664698 10.1016/j.omtn.2022.04.011 PMC9130092

[36] Wang W Luo J Wang S. Recent progress in isolation and detection of extracellular vesicles for cancer diagnostics. Adv Healthcare Mater. 2018;7:e1800484. 10.1002/adhm.201800484 30009550

[37] Islam MS Gopalan V Lam AK Shiddiky MJA. Current advances in detecting genetic and epigenetic biomarkers of colorectal cancer. Biosens Bioelectron. 2023;239:115611. 37619478 10.1016/j.bios.2023.115611

[38] Aboulkheyr Es H Montazeri L Aref AR Vosough M Baharvand H. Personalized cancer medicine: an organoid approach. Trends Biotechnol. 2018;36:358–71. 29366522 10.1016/j.tibtech.2017.12.005

[39] Panesar S Neethirajan S. Microfluidics: rapid diagnosis for breast cancer. Nano Micro Letters. 2016;8:204–20. 30460281 10.1007/s40820-015-0079-8 PMC6223681

[40] Sukanya VS Rath SN. Microfluidic biosensor-based devices for rapid diagnosis and effective anti-cancer therapeutic monitoring for breast cancer metastasis. Adv Exp Med Biol. 2022;1379:319–39. 35760998 10.1007/978-3-031-04039-9_13

[41] Neoh KH Hassan AA Chen A. Rethinking liquid biopsy: microfluidic assays for mobile tumor cells in human body fluids. Biomaterials. 2018;150:112–24. 29035737 10.1016/j.biomaterials.2017.10.006

[42] Mohammadi M Zargartalebi H Salahandish R Aburashed R Yong KW Sanati-Nezhad A. Emerging technologies and commercial products in exosome-based cancer diagnosis and prognosis. Biosens Bioelectron. 2021;183:113176. 33845291 10.1016/j.bios.2021.113176

[43] Jackson JM Witek MA Kamande JW Soper SA. Materials and microfluidics: enabling the efficient isolation and analysis of circulating tumour cells. Chem Soc Rev. 2017;46:4245–80. 28632258 10.1039/c7cs00016b PMC5576189

[44] Khorsandi D Yang JW Foster S. Patient-derived organoids as therapy screening platforms in cancer patients. Adv Healthcare Mater. 2024;13:e2302331. 10.1002/adhm.202302331 PMC11324859 38359321

[45] Rupp B Ball H Wuchu F Nagrath D Nagrath S. Circulating tumor cells in precision medicine: challenges and opportunities. Trends Pharmacol Sci. 2022;43:378–91. 35272862 10.1016/j.tips.2022.02.005

[46] Zhang Y Duan Z Shu P Deng J. Exploring acceptable risk in engineering and operations research and management science by bibliometric analysis. Risk Analys. 2023;43:1539–56. 10.1111/risa.14049 36307897

[47] Song L Zhang J Ma D. A Bibliometric and knowledge-map analysis of macrophage polarization in atherosclerosis from 2001 to 2021. Front Immunol. 2022;13:910444. 35795675 10.3389/fimmu.2022.910444 PMC9250973

[48] Wu PN Liu JL Fang MJ. Global trends in colorectal cancer and metabolic syndrome research: a bibliometric and visualization analysis. Int J Surg (London, England). 2024;110:3723–33. 10.1097/JS9.0000000000001342 PMC11175816 38498393

