# Supplementary material for: Mapping the evolution and impact of microfluidic technology research on cancer diagnosis: A comprehensive bibliometric analysis from 2015 to 2024
Source: Medicine (Baltimore). 2026 Jul 31;105(31):e49910. doi: 10.1097/MD.0000000000049910 (PMC13433093; doi:10.1097/MD.0000000000049910)
Supplement: Supplementary file 1 [file medi-105-e49910-s001.docx]

| **References** | **Title** |
| --- | --- |
| Sollier E, 2014, LAB CHIP, V14, P63, DOI 10.1039/c3lc50689d | Size-selective collection of circulating tumor cells using Vortex technology |
| Ozkumur E, 2013, SCI TRANSL MED, V5, P0, DOI 10.1126/scitranslmed.3005616 | Inertial Focusing for Tumor Antigen–Dependent and –Independent Sorting of Rare Circulating Tumor Cells |
| Sarioglu AF, 2015, NAT METHODS, V12, P685,  DOI 10.1038/NMETH.3404 | A microfluidic device for label-free, physical capture of circulating tumor cell clusters |
| Alix-Panabières C, 2013, CLIN CHEM, V59, P110,  DOI 10.1373/clinchem.2012.194258 | Circulating Tumor Cells: Liquid Biopsy of Cancer |
| Karabacak NM, 2014, NAT PROTOC, V9, P694,  DOI 10.1038/nprot.2014.044 | Microfluidic, marker-free isolation of circulating tumor cells from blood samples |
| Hou HW, 2013, SCI REP-UK, V3, P0, DOI  10.1038/srep01259 | Isolation and retrieval of circulating tumor cells using centrifugal forces |
| Warkiani ME, 2016, NAT PROTOC, V11, P134, DOI  10.1038/nprot.2016.003 | Ultra-fast, label-free isolation of circulating tumor cells from blood using spiral microfluidics |
| Chen YC, 2014, LAB CHIP, V14, P626, DOI  10.1039/c3lc90136j | Rare cell isolation and analysis in microfluidics |
| Sackmann EK, 2014, NATURE, V507, P181, DOI  10.1038/nature13118 | The present and future role of microfluidics in biomedical research |
| Warkiani ME, 2014, LAB CHIP, V14, P128, DOI 10.1039/c3lc50617g | Slanted spiral microfluidics for the ultra-fast, label-free isolation of circulating tumor cells |
| Li P, 2015, P NATL ACAD SCI USA, V112, P4970,  DOI 10.1073/pnas.1504484112 | Acoustic separation of circulating tumor cells |
| Zhao Z, 2016, LAB CHIP, V16, P489, DOI  10.1039/c5lc01117e | A microfluidic ExoSearch chip for multiplexed exosome detection towards blood-based ovarian cancer diagnosis |
| Kanwar SS, 2014, LAB CHIP, V14, P1891, DOI  10.1039/c4lc00136b | Microfluidic device (ExoChip) for on-chip isolation, quantification and characterization of circulating exosomes |
| Wunsch BH, 2016, NAT NANOTECHNOL, V11,  P936, DOI 10.1038/nnano.2016.134 | Nanoscale lateral displacement arrays for the separation of exosomes and colloids down to 20 nm |
| Zhang P, 2016, LAB CHIP, V16, P3033, DOI  10.1039/c6lc00279j | Ultrasensitive microfluidic analysis of circulating exosomes using a nanostructured graphene oxide/polydopamine coating |
| Bray Freddie, 2018, CA CANCER J CLIN, V68,  P394, DOI 10.3322/caac.21609 | Erratum: Global cancer statistics 2018: GLOBOCAN estimates of incidence and mortality worldwide for 36 cancers in 185 countries |
| Xu HY, 2018, ANAL CHEM, V90, P13451, DOI  10.1021/acs.analchem.8b03272 | Magnetic-Based Microfluidic Device for On-Chip Isolation and Detection of Tumor-Derived Exosomes |
| Zhang P, 2019, NAT BIOMED ENG, V3, P438, DOI  10.1038/s41551-019-0356-9 | Ultrasensitive detection of circulating exosomes with a 3D-nanopatterned microfluidic chip |
| van Niel G, 2018, NAT REV MOL CELL BIO, V19,  P213, DOI 10.1038/nrm.2017.125 | Shedding light on the cell biology of extracellular vesicles |
| Belotti Y, 2021, ANAL CHEM, V93, P4727, DOI  10.1021/acs.analchem.1c00410 | Microfluidics for Liquid Biopsies: Recent Advances, Current Challenges, and Future Directions |
| Sung H, 2021, CA-CANCER J CLIN, V71, P209, DOI  10.3322/caac.21660 | Global Cancer Statistics 2020: GLOBOCAN Estimates of Incidence and Mortality Worldwide for 36 Cancers in 185 Countries |
| Lin DF, 2021, SIGNAL TRANSDUCT TAR, V6, P0, DOI 10.1038/s41392-021-00817-8 | Circulating tumor cells: biology and clinical significance |
| Lin BQ, 2021, SMALL METHODS, V5, P0, DOI  10.1002/smtd.202001131 | Microfluidic-Based Exosome Analysis for Liquid Biopsy |
| Niculescu AG, 2021, INT J MOL SCI, V22, P0, DOI  10.3390/ijms22042011 | Fabrication and Applications of Microfluidic Devices: A Review |
| Yu D, 2022, MOL CANCER, V21, P0, DOI  10.1186/s12943-022-01509-9 | Exosomes as a new frontier of cancer liquid biopsy |
