# Supplementary material for: Mapping the evolution and impact of microfluidic technology research on cancer diagnosis: A comprehensive bibliometric analysis from 2015 to 2024
Source: Medicine (Baltimore). 2026 Jul 31;105(31):e49910. doi: 10.1097/MD.0000000000049910 (PMC13433093; doi:10.1097/MD.0000000000049910)
Supplement: Supplementary file 2 [file medi-105-e49910-s002.docx]

Cluster 1 (17)

amplification

biomarker

biomarkers

biosensor

biosensors

breast cancer

cells

diagnosis

dna

gold nanoparticles

immunoassay

microfluidic

microfluidic chip

nanoparticles

platform

quantification

sensitive detection

Cluster 2 (14)

| blood  cancer - cells  capture  chip  circulating tumor cells  circulating tumor - cells  device  dielectrophoresis  enrichment  label - free isolation  separation  size  system  whole – blood  Cluster 3 (8)  breast - cancer  exosomes  extracellular vesicles  label - free  liquid biopsy  lung - cancer  peripheral - blood  prostate - cancer |
| --- |

Cluster 4 (5)

expression

in - vitro

metastasis

microfluidic device

on - a - chip
